# Bullying and oral health in Egyptian adolescents: the moderating role of sense of coherence and resistance to peer influence

**DOI:** 10.1186/s12903-024-04937-9

**Published:** 2024-10-18

**Authors:** Amira H. Elwan, Abdelwahab Samaha, Wafaa E. Abdelaziz

**Affiliations:** Department of Pediatric Dentistry and Dental Public Health, Faculty of Dentistry, Alexandria University, Alexandria, Egypt

**Keywords:** Oral health, Caries, Gingival inflammation, Toothbrushing, Bullying, Sense of coherence, Resistance to peer influence

## Abstract

**Background:**

Bullying is the intentional, repeated and prolonged aggressive behavior towards victim(s) who feel powerless to defend themselves. It could influence adolescents’ mental health. Some adolescents possess coping skills which enable them to overcome such adversities. The present study assessed the association between bullying, sense of coherence (SOC), resistance to peer influence (RPI) and oral health in Egyptian adolescents.

**Materials and methods:**

A cross-sectional survey that included 12–16 year old adolescents attending 6 schools in Damanhour, Egypt was conducted from March to December 2023. Data were collected through clinical examination and self-administered questionnaires. Clinical examination assessed dental caries (DMFT), oral hygiene (plaque index) and gingival condition (gingival index). Toothbrushing frequency and sugar consumption were assessed by (World Health Organization-child form), bullying (Revised Olweus Bully Victim Questionnaire), SOC (Sense of Coherence Scale-Short Form) and RPI (Resistance to Peer Influence Questionnaire). The independent variables were bullying, SOC and RPI. The dependent variables were untreated caries, gingival inflammation and toothbrushing frequency. Multivariable multilevel regression assessed the relationship between the independent and dependent variables after adjusting for potential confounders. Adjusted regression coefficients (B), adjusted odds ratios (AOR) and 95% confidence intervals (CI) were calculated. Effect modifications by SOC and RPI were evaluated.

**Results:**

The response rate was 95.25% (*N* = 602), mean (SD) age was 14.01 (1.15). Half of the students (49.83%) were girls. About 42% had untreated caries, only 15.78% brushed their teeth twice daily and the mean (SD) gingival index was 1.57 (0.40). About 25% were victims, (9.30%) bullies and (18.60%) bully-victims. Victims showed significantly lower odds of twice daily toothbrushing (AOR = 0.52, *p* = 0.04). Bullies and bully-victims showed non-significantly higher odds of untreated caries (AOR = 1.42, *p* = 0.25), (AOR = 1.21, *p* = 0.42), respectively. Bullying was not associated with gingival inflammation. Higher SOC and RPI mitigated the impact of bullying on untreated caries and toothbrushing frequency.

**Conclusion:**

Bullying was associated with higher odds of untreated caries and lower odds of twice daily toothbrushing in Egyptian adolescents. Higher SOC and RPI alleviated this relationship, highlighting the importance of individual coping strategies to oral health.

**Supplementary Information:**

The online version contains supplementary material available at 10.1186/s12903-024-04937-9.

## Background

Oral health is an integral component of general health and a principal determinant of the quality of life [1]. The World Health Organization (WHO) defines adolescence as the period between childhood and adulthood, from ages 10 to 19 [2]. It is considered an essential period for treating oral disease [3] and establishing oral health promoting behaviors [4]. However, this could be challenging as adolescents undergo physical and emotional changes [5, 6]. Elevated sex hormones could lead to puberty gingivitis [7]. Emotional distress is usually associated with a higher likelihood of periodontal disease [8], dental caries [9], recurrent aphthous ulcers [10], temporomandibular pain disorders [11] and bruxism [12]. Additionally, they begin to gain autonomy over individual health behavior [13]. For instance, they tend to brush their teeth infrequently [14] and consume sugar-rich food and soft drinks [15, 16]. This would induce dental caries, periodontal disease and tooth erosion [17, 18]. They might also face direct aggression from peers which may result in traumatic orofacial injuries [19] and/or indirect aggression, such as bullying [20]. Bullying is considered the most common form of conflict in adolescence [21]. Olweus [22] defined school bullying as the intentional, repeated and prolonged exposure to negative actions by one or more students with an imbalance of power between the bully and victim. Individuals who both bully others and are bullied themselves are defined as bully-victims [23].

Bullying is associated with a myriad of psychological outcomes. Adolescents involved in bullying could suffer from stress, anxiety and depression [24–26]. While victims tend to demonstrate social withdrawal [27], low self-esteem [28], loneliness [29] and suicidal ideation [30], bullies are more likely to show conduct problems and antisocial behavior [31, 32]. Stress and depression are key risk factors of dental caries [33, 34] and gingival inflammation [35], because psychological distress could compromise the host resistance to oral bacteria [36], reduce salivary flow [37, 38] and drive individuals to adopt unhealthy practices, including decreased toothbrushing [39], frequent snacking of carbohydrates [40] and using psychoactive substances [41–43].

Previous studies reported a significant association between verbal bullying victimization, untreated caries and caries complications in children [44, 45]. A Brazilian study found that children who experienced verbal bullying victimization had an increased odds of having untreated caries and pulp exposure [46]. In adolescents, a Norwegian study reported that bullying victimization was significantly associated with untreated caries, yet the study analyzed data from dental health records generated by multiple examiners whose calibration was not optimal and their reliability values were omitted [47]. A Nigerian study, on the other hand, found no association between bullying victimization and untreated caries or oral hygiene. However, the study was conducted in a community with remarkably low caries prevalence that its results cannot be generalized [48]. An Australian study also showed no significant association between bullying victimization and self-reported dental problems among indigenous adolescents [49], who are considered a very culturally distinct population [50], so their findings cannot be generalized either. None of the previous studies investigated the impact of bullying perpetration or perpetration-victimization on oral health.

Kochenderfer-Ladd et al. [51] suggested that features of bullying should be studied while taking the individual characteristics into account as they could explain why bullying places some students at greater risk while some adapt. Similarly, Fisher-owens et al. [52] showed that some people possess individual coping skills which enable them to overcome adversities, thus protect them against poor oral health outcomes. Two of these skills are: sense of coherence (SOC) [53] and resistance to peer influence (RPI) [54].

Antonovsky [53] devised the salutogenic model of health to explain the correlation between stress, coping and health. SOC, the central construct of the salutogenic model, consists of three components (comprehensibility, manageability and meaningfulness). It expresses the extent to which one has a feeling of confidence that they can understand (comprehensibility), handle (manageability) and make sense (meaningfulness) of an experience or disease. The higher the SOC, the higher the individual’s capability to successfully cope with the experience or disease [55]. Adolescents with a weaker SOC are more likely to fall victims to bullying [56]. A meta-analysis showed that SOC is significantly associated with caries rate and toothbrushing frequency among adults, yet the number of articles available to establish such a relationship among adolescents is considered insufficient [57]. RPI represents the extent to which an individual acts autonomously while interacting with peers [58], thus RPI influences how far an individual adopts their peers’ normative and health behavior [59, 60]. The association between RPI and oral health is yet to be explored. SOC and RPI could have a moderating effect on the relationship between bullying and oral health.

According to The United Nations Educational, Scientific and Cultural Organization (UNESCO), 32% of the globe’s adolescents experience bullying with the highest prevalence in North Africa and the Middle East [61]. However, the evidence on the relationship between bullying and oral health in these regions is scarce. The differences between the Eastern and Western perspectives play a major role in the bullying dynamics and the related coping strategies [62], which could largely influence the associated oral health outcomes and accentuate the already-existing oral health disparities. Egypt is a developing middle eastern country located in the northeastern corner of Africa. It is the most populous Arab country with a population of over 100 million people [63]. Adolescents represent 19% of its population [64]. Student Health Surveys report that up to 79% of Egyptian adolescents are victims of bullying [21].

This study aimed to explore the association between bullying, SOC and RPI on one hand, and oral health in Egyptian adolescents on the other hand. The study findings would help identify the psychosocial factors associated with oral health in adolescents living in developing countries, which could be useful in planning public strategies aiming at enhancing their oral health. The null hypothesis was that there would be no significant association between bullying, SOC, RPI and oral health in Egyptian adolescents.

## Materials and methods

A cross-sectional survey was conducted in Damanhour (the capital of Elbeheira governorate) in Northwestern Nile-Delta in Egypt. Data were collected from March to December 2023. Prior to the study, the ethical approval was obtained from the Research Ethics Committee at The Faculty of Dentistry, Alexandria University, Egypt (IORG0008839–0630 – 02/2023). Permissions from The Central Agency for Public Mobilization and Statistics (CAPMAS) and the local government’s authorities of education were secured. Written informed consents from the guardians of eligible students and students’ assent were obtained. In the consent form, guardians were asked to provide the mothers’ highest educational level as an indicator of their socioeconomic status.

### Sampling

Limited number of students were enrolled in private schools, so no data was collected from these schools and no stratification was done by public versus private school status. Multistage cluster sampling was carried out. In the first stage: public schools were divided into three strata according to district. At the second stage, each stratum was further divided into all-boys and all-girls schools. Two schools were randomly selected from each district, one all-boys school and one all-girls school. Thus, data was collected from 6 public schools. In each school, all attending adolescents were included in the study, if they fit the inclusion criteria. Students were considered eligible if they were aged 12 to 16 and living in Damanhour city. Intellectually disabled students and those who did not provide parental consent were excluded from the study.

### Sample size

Sample size was calculated assuming a 95% confidence interval and 80% power to detect the relation between bullying and untreated caries. Duarte-Rodigues et al. [46] reported that students who had untreated dental caries had a higher likelihood of bullying, odds ratio = 1.76. For a logistic regression analysis, the required sample size was calculated to be 164, increased to 180 to compensate for 10% non-response rate. Assuming that the intraclass correlation coefficient (ICC) within schools = 0.03 [65] and 80 students will be included from each cluster, the design effect would be 3.37 [66]. The required sample size to accommodate the clustering effect = sample size x design effect = 180 × 3.37 = 606.7 ≈ 607 adolescents [66, 67].

### Data collection

Data were collected through an anonymous self-administered questionnaire and clinical examination. The Arabic validated version [68] of the WHO questionnaire child form [69] was used to collect information about students’ background: (age and sex) and oral health behaviors, including toothbrushing frequency with a fluoridated toothpaste (two or more times per day or less) and sugar consumption (at least once daily or less of 8 types of sugary foods/snacks: fruits, biscuits or cakes, carbonated beverages or lemon juice, jam or honey, sugar-added chewing gums, candy, sugar-sweetened milk and sugar-sweetened hot beverages). The sugar consumption score was created by summing the number of products which were consumed at least once daily. The scores ranged from 0 (lowest) to 8 (highest) sugar consumption.

*The Revised Olweus Bully Victim questionnaire (OBVQ-R)* [70] *was used to assess the prevalence of bullying. A detailed explanation of bullying was given to students then they were asked to answer the questionnaire. The OBVQ-R is divided into two sets of questions (Bullying victimization and perpetration). For bullying victimization*,* it provides a global question about the frequency he/she was bullied during the past couple of months. This is followed by 8 specific questions asking on the frequency of the occurrence of specific types of bullying: (verbal*,* physical*,* social exclusion*,* rumors*,* personal things taken away*,* threatening*,* color*,* cyberbullying and other). The question about sexual bullying was omitted due to cultural reasons*,* as well as the question about racial bullying because Egyptians are a fairly homogeneous population who share the same race* [71, 72]. *For bullying perpetration*,* the OBVQ-R provides corresponding global and specific questions on the frequency he/she bullied another student(s). All questions are rated on a 4-point Likert scale (It hasn’t happened in the past couple of months*,* it has only happened once or twice*,* 2 or 3 times a month*,* once or several times a week). For a student to be denoted as a victim*,* he/she must answer (2 or 3 times a month or once or several times a week) to the victimization global question or any of the specific victimization questions. For a student to be denoted as a perpetrator*,* he/she must answer (2 or 3 times a month or once or several times a week) to the perpetration global question or any of the specific perpetration questions. A student was categorized as bully-victim if he/she answered (2 or 3 times a month or once or several times a week) to both global questions or answered (2 or 3 times a month or once or several times a week) to any specific victimization question as well as any specific perpetration question. Otherwise*,* the student was categorized as not-involved* [73].

*The Sense of Coherence Short Form Scale (SOC-13)* [74] *was used to assess the level of SOC. It has 13 items which measure the three SOC components: comprehensibility (5 items)*,* manageability (4 items) and meaningfulness (4 items). Each item is rated on a 7-point Likert scale and 5 items require reverse scoring. The SOC total score is calculated by summing scores of all items and ranges between 13 (lowest) to 91 (highest) SOC.*

*Resistance to Peer Influence Questionnaire (RPIQ)* [58] *was used to measure the level of RPI. It has 10 items which require students to choose the statements which best describes the group of people they belong to (less vs. more peer-resistant) then they were asked to indicate to what extent they feel they belong to the chosen group (“Really true” vs. “Sort of true”). Responses to each two subitems were aggregated on a 4-point Likert scale where the “Really true” and “Sort of true” choice of the less peer-resistant statements were coded as 1 and 2*,* respectively. The “Sort of true” and “Really true” choice of the more peer-resistant statements were coded as 3 and 4*,* respectively. Three items require reverse scoring. The total score was calculated by summing the scores of all items and ranges between 10 (lowest) and 40 (highest) resistance.*

The three scales were translated to Arabic and back translated to English to ensure matching and validity. Five experts assessed their face and content validity [72]. The mean content validity index for the OBVQ-R, SOC-13, and RPIQ was 0.979, 0.985 and 0.981, respectively. The questionnaires were pilot tested on 30 students from a random school to ensure the comprehensibility of the questionnaire and assess the time needed for responding. Their data were excluded from the final analysis.

Dental caries was assessed using the DMFT index according to the WHO criteria, where D = Decayed, M = Missing, F = Filled teeth [69]. A single examiner A.H.E. was calibrated with a gold standard examiner. The inter-examiner reliability was tested and intra-examiner reliability was tested after 1 and 2 weeks (Mean Kappa score = 0.90), which indicated excellent agreement within the examiner across time [75]. The gingival condition was assessed using the gingival index of Löe and Silness (GI) [76] Oral hygiene was measured by the plaque index of Silness and Löe (PLI) [77] (Appendix I). Intraoral assessment was done in the presence of a school supervisor. The examiner used sterilizable mirrors and ball-ended WHO probes #550B under daylight without magnification or drying. Students were referred for treatment if needed. All students received toothpaste and toothbrushes with health education instructions.

### Statistical analysis

The independent variables (IDV) were bullying, SOC and RPI. The dependent variables (DV) were untreated caries, gingival inflammation and toothbrushing frequency. Normality of the quantitative variables was tested. The association between the IDV and DV were tested regarding age, sex, mother’s education, toothbrushing frequency, sugar consumption score, plaque index, bullying, SOC and RPI using independent samples t-test, Chi square test, Mann-Whitney U test or ANOVA based on the number of groups or whether they were normally distributed.

Multivariable multilevel binary logistic regression analysis was used to assess the relationship between the IDVs, untreated caries and toothbrushing frequency. Multivariable multilevel linear regression was used to assess the relationship between the IDVs and gingival inflammation, controlling for potential confounders (age, sex, mother’s education, toothbrushing frequency, sugar consumption score and plaque index). Plaque index and toothbrushing frequency were not adjusted for in the toothbrushing frequency model. The interactions of SOC and RPI on bullying were assessed. Adjusted odds ratios (AOR), adjusted regression coefficients (B), 95% confidence intervals (CI) were calculated. Significance level was set at *p* < 0.05. Data was analyzed using IBM SPSS Version 23 for Windows (SPSS Inc., Chicago, USA).

## Results

**Fig. 1:**
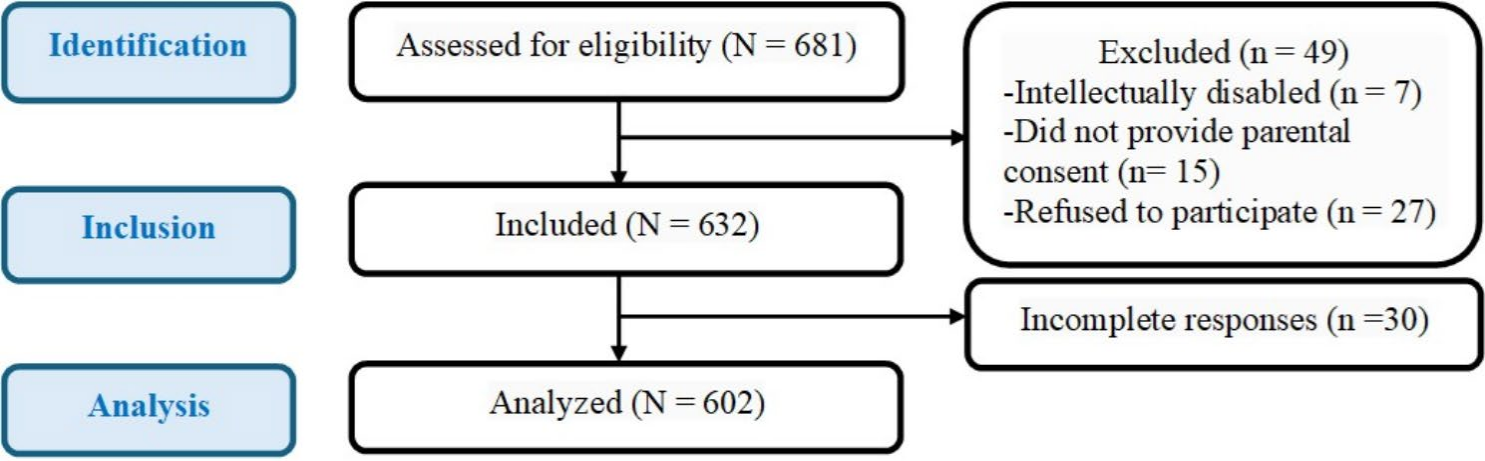
Flow chart showing the flow of participants in the study

A total of 602 students returned complete questionnaires (response rate = 95.25%) **(**Fig. 1**)**. Boys represented half of the study sample (50.17%). The mean ± SD age in years = 14.01 ± 1.15. More than half of the mothers had university education or above (53.49%). Only 15.78% brushed their teeth twice daily. The Median (IQR) of sugar consumption score and plaque index were 6 (5, 7) and 2.17 (2, 2.33), respectively. Over half the students (52.48%) were involved in bullying; (24.58%) victims, (9.30%) bullies and (18.60%) bully-victims. The mean ± SD SOC and RPI scores = 51.82 ± 13.25 and 25.30 ± 4.41, respectively. Untreated caries accounted for 41.53% and the mean ± SD gingival index was 1.57 ± 0.40. **(**Table 1**)**.

**Table 1:** Description of the study participants (*N* = 602)

Age in years	Mean ± SD	14.01 ± 1.15
Sex	Girls N (%)	300 (49.83)
	Boys N (%)	302 (50.17)
Mother’s educational level	University or above N (%)	322 (53.49)
	Below university N (%)	280 (46.51)
Toothbrushing frequency	Twice or more a day N (%)	95 (15.78)
	Less than twice a day N (%)	507 (84.22)
Sugar consumption score^1^	Median (IQR)	6 (5, 7)
Bullying	Victims N (%)	148 (24.58)
	Bullies N (%)	56 (9.30)
	Bully-victims N (%)	112 (18.60)
	Not involved N (%)	286 (47.51)
SOC score^2^	Mean ± SD	51.82 ± 13.25
RPI score^3^	Mean ± SD	25.30 ± 4.41
Untreated dental caries in permanent teeth (DT)	N (%)	250 (41.53)
	Mean ± SD	1.03 ± 1.65
	Median (IQR)	0.00 (0.00, 2.00)
Gingival index^4^	Mean ± SD	1.57 ± 0.40
Plaque index^5^	Median (IQR)	2.17 (2, 2.33)

*SD* Standard Deviation, *IQR* Interquartile range, *SOC* Sense of coherence, *RPI* Resistance to peer influence

Scores were calculated as: 1. (0) to (8) highest sugar consumption, 2. (13) to (91) highest SOC, 3. (10) to (40) highest RPI, 4. (0) to (3) severe inflammation, 5. (0) to (3) severe accumulation of plaque.

Bullying, SOC and RPI showed no significant association with untreated caries, gingival inflammation or toothbrushing frequency, except for SOC which was significantly associated with twice daily toothbrushing (*p* = 0.03), where the higher the SOC score, the higher the twice daily toothbrushing **(**Table 2**)**.

**Table 2:** Bivariate analysis of the relationship between the independent variables, untreated caries, gingival inflammation and toothbrushing frequency in the participants (*N* = 602)

Independent variables		Teeth with untreated caries *N* (%)			Gingival index		Toothbrushing frequency *N* (%)		
		Decayed	Not Decayed	*p* value	-	*p* value	Twice or more a day	Less than twice a day	*p* value
Age in years	Mean ± SD	13.96 ± 1.16	14.04 ± 1.14	0.41 ^a^	-	-	14.07 ± 1.12	14.00 ± 1.15	0.56 ^a^
Sex	Girls N (%)	134 (53.60)	166 (47.16)	0.12 ^b^	1.54 ± 0.39	0.11 ^a^	51 (53.68)	249 (49.11)	0.41 ^b^
	Boys N (%)	116 (46.40)	186 (52.84)		1.59 ± 0.42		44 (46.32)	258 (50.89)	
Mother’s educational level	University or above N (%)	131 (52.40)	191 (54.26)	0.65 ^b^	1.56 ± 0.42	0.11 ^a^	56 (58.95)	266 (52.47)	0.25 ^b^
	Below University N (%)	119 (47.60)	161 (45.74)		1.57 ± 0.39		39 (41.05)	241 (47.53)	
Toothbrushing frequency	Twice or more a day N (%)	42 (16.80)	53 (15.06)	0.56 ^b^	1.51 ± 0.41	0.17 ^a^	-	-	-
	Less than twice a day N (%)	208 (83.20)	299 (84.94)		1.58 ± 0.40		-	-	-
Sugar consumption score	Median (IQR)	6 (5, 7)	6 (5, 7)	0.32 ^c^	-	-	6 (5, 7)	6 (5, 7)	0.48 ^c^
Plaque index	Median (IQR)	2.17 (2, 2.33)	2.17 (2, 2.33)	0.06 ^c^	-	-	2.17 (2, 2.33)	2.17 (2, 2.33)	0.06 ^c^
Bullying	Victims N (%)	60 (24.00)	88 (25.00)	0.57 ^b^	1.53 ± 0.43	0.51 ^d^	15 (15.79)	133 (26.23)	0.05 ^b^
	Bullies N (%)	27 (10.80)	29 (8.24)		1.58 ± 0.43		6 (6.32)	50 (9.86)	
	Bully-victims N (%)	50 (20.00)	62 (17.61)		1.60 ± 0.38		18 (18.95)	94 (18.54)	
	Not involved N (%)	113 (45.20)	173 (49.15)		1.57 ± 0.39		56 (58.95)	230 (45.36)	
SOC score	Mean ± SD	51.71 ± 14.16	51.90 ± 12.57	0.86 ^a^	-	-	54.49 ± 13.05	51.32 ± 13.23	0.03* ^a^
RPI score	Mean ± SD	25.17 ± 4.31	25.40 ± 4.49	0.53 ^a^	-	-	25.87 ± 4.57	25.20 ± 4.38	0.17 ^a^

*SD* Standard Deviation, *IQR* Interquartile range, *SOC* Sense of coherence, *RPI* Resistance to peer influence

^a^Independent t-test, ^b^Chi square test, ^c^Mann Whitney U test, ^d^ANOVA test

*Statistically significant at *p* < 0.05

In multivariable multilevel logistic regression, bullies and bully-victims showed non-significantly higher odds of untreated caries (AOR = 1.42, 95% CI = 0.79, 2.56) and (AOR = 1.21, 95% CI = 0.76, 1.92), respectively. SOC and RPI were not associated with untreated caries (AOR = 1.003, 95% CI = 0.99, 1.02) and (AOR = 0.99, 95% CI = 0.95, 1.03), respectively.

SOC and RPI non-significantly modified the association between bullying perpetration and untreated caries (AOR = 1.01, 95% CI = 0.99, 1.02) and (AOR = 1.01, 95% CI = 0.99, 1.04), respectively, and non-significantly modified this association for bullying perpetration-victimization (AOR = 1.002, 95% CI = 0.99, 1.01) and (AOR = 1.01, 95% CI = 0.99, 1.02), respectively **(**Table 3**)**.

**Table 3:** Multivariable multilevel logistic regression of the association between bullying, sense of coherence, resistance to peer influence and untreated caries in the participants (*N* = 602)

Explanatory variables		AOR (95% CI)	*p* value
Age		0.97 (0.83, 1.12)	0.65
Sex	Girls	1.37 (0.97, 1.93)	0.08
	Boys	Reference category	
Mother’s educational level	Above university	0.97 (0.69, 1.36)	0.84
	Below University	Reference category	
Toothbrushing frequency	Twice or more a day	1.20 (0.76, 1.87)	0.45
	Less than twice a day	Reference category	
Sugar consumption score		1.08 (0.97, 1.19)	0.18
Plaque index		1.51 (0.96, 2.37)	0.07
Bullying	Victims	0.98 (0.64, 1.51)	0.93
	Bullies	1.42 (0.79, 2.56)	0.25
	Bully-victims	1.21 (0.76, 1.92)	0.42
	Not involved	Reference category	
SOC score		1.003 (0.99, 1.02)	0.70
RPI score		0.99 (0.95, 1.03)	0.53
**Interaction**			
SOC x Victims		0.999 (0.98, 1.01)	0.83
SOC x Bullies		1.01 (0.99, 1.02)	0.16
SOC x Bully-victims		1.002 (0.99, 1.01)	0.60
SOC x Not involved		Reference category	
RPI x Victims		0.999 (0.98, 1.02)	0.94
RPI x Bullies		1.01 (0.99, 1.04)	0.32
RPI x Bully-victims		1.01 (0.99, 1.02)	0.54
RPI x Not involved		Reference category	

*AOR* adjusted odds ratio, *CI* confidence interval, *SOC* Sense of coherence, *RPI* Resistance to peer influence

Adjusted for age, sex, mother’s educational level, toothbrushing frequency, sugar consumption score and plaque index

*Statistically significant at *p* < 0.05

In multivariable multilevel linear regression, bullying victimization, perpetration, and perpetration-victimization were not associated with gingival inflammation (B = -0.05, 95% CI = -0.13, 0.02), (B = -0.02, 95% CI = -0.13, 0.08) and (B = -0.01, 95% CI = -0.09, 0.07), respectively. SOC and RPI had no association with gingival inflammation (B = -0.001, 95% CI = -0.003, 0.001) and (B = 0.003, 95% CI = -0.004, 0.01), respectively. The interaction between bullying, SOC and RPI had no influence on the relationship with gingival inflammation **(**Table 4**)**.

**Table 4:** Multivariable multilevel logistic regression of the association between bullying, sense of coherence, resistance to peer influence, and gingival inflammation in the participants (*N* = 602)

Explanatory variables		B (95% CI)	*p* value
Age		-0.003 (-0.04, 0.03)	0.85
Sex	Girls	-0.05 (-0.13, 0.03)	0.23
	Boys	Reference category	
Mother’s educational level	Above university	-0.03 (-0.10, 0.04)	0.39
	Below University	Reference category	
Toothbrushing frequency	Twice or more a day	0.02 (-0.10, 0.06)	0.57
	Less than twice a day	Reference category	
Sugar consumption score		0.001 (-0.02, 0.02)	0.93
Plaque index		0.49 (0.41, 0.57)	< 0.001*
Bullying	Victims	-0.05 (-0.13, 0.02)	0.17
	Bullies	-0.02 (-0.13, 0.08)	0.68
	Bully-victims	-0.01 (-0.09, 0.07)	0.79
	Not involved	Reference category	
SOC score		-0.001 (-0.003, 0.001)	0.37
RPI score		0.003 (-0.004, 0.01)	0.34
**Interaction**			
SOC x Victims		-0.001 (-0.002, 0.001)	0.22
SOC x Bullies		-0.000 (-0.003, 0.002)	0.66
SOC x Bully-victims		-0.000 (-0.002, 0.001)	0.83
SOC x Not involved		Reference category	
RPI x Victims		-0.002 (-0.005, 0.001)	0.24
RPI x Bullies		-0.001 (-0.005, 0.003)	0.59
RPI x Bully-victims		-0.001 (-0.004, 0.002)	0.62
RPI x Not involved		Reference category	

*B* standardized regression coefficient, *CI* confidence interval, *SOC* Sense of coherence, *RPI* Resistance to peer influence

Adjusted for age, sex, mother’s educational level, toothbrushing frequency, sugar consumption score and plaque index

*Statistically significant at *p* < 0.05

In multivariable multilevel logistic regression, victims showed significantly lower odds of twice daily toothbrushing (AOR = 0.52, 95% CI = 0.28, 0.99), and bullies had non-significantly lower odds of twice daily toothbrushing (AOR = 0.57, 95% CI = 0.23, 1.43). SOC and RPI had no association with toothbrushing frequency (AOR = 1.02, 95% CI = 0.997, 1.03) and (AOR = 1.01, 95% CI = 0.96, 1.07), respectively.

SOC non-significantly modified the association between bullying victimization and toothbrushing frequency (AOR = 0.99, 95% CI = 0.98, 0.999), while RPI significantly modified this association (AOR = 0.97, 95% CI = 0.94, 0.99). SOC and RPI non-significantly modified this association between bullying perpetration and toothbrushing frequency (AOR = 0.99, 95% CI = 0.97, 1.01) and (AOR = 0.98, 95% CI = 0.95, 1.01), respectively **(**Table 5**)**.

**Table 5:** Multivariable multilevel logistic regression for the association between bullying, sense of coherence, resistance to peer influence, and toothbrushing frequency in the participants (*N* = 602)

Explanatory variables		AOR (95% CI)	*p* value
Age		0.04 (0.84, 1.)	0.77
Sex	Girls	1.03 (0.90, 2.36)	0.13
	Boys	Reference category	
Mother’s educational level	Above university	1.46 (0.76, 1.98)	0.40
	Below University	Reference category	
Sugar consumption score		0.97 (0.84, 1.12)	0.66
Bullying	Victims	0.52 (0.28, 0.99)	0.04*
	Bullies	0.57 (0.23, 1.43)	0.23
	Bully-victims	0.93 (0.50, 1.71)	0.80
	Not involved	Reference category	
SOC score		1.02 (0.997, 1.03)	0.11
RPI score		1.01 (0.96, 1.07)	0.66
**Interaction**			
SOC x Victims		0.99 (0.98, 0.999)	0.06
SOC x Bullies		0.99 (0.97, 1.01)	0.33
SOC x Bully-victims		1.00 (0.99, 1.01)	0.94
SOC x Not involved		Reference category	
RPI x Victims		0.97 (0.94, 0.99)	0.01*
RPI x Bullies		0.98 (0.95, 1.01)	0.80
RPI x Bully-victims		0.995 (0.97, 1.02)	0.65
RPI x Not involved		Reference category	

*AOR* adjusted odds ratio, *CI* confidence interval, *SOC* Sense of coherence, *RPI* Resistance to peer influence

Adjusted for age, sex, mother’s educational level and sugar consumption score

*Statistically significant at *p* < 0.05

## Discussion

Findings of the current study showed that over half of the 12–16 year old adolescents in public middle schools of Egypt were involved in bullying. Less than half of the students had untreated caries, the gingival inflammation was moderate and the majority brushed their teeth less than twice daily. There was no significant association between bullying perpetration, perpetration-victimization, SOC, RPI and oral health. However, bullying victimization significantly decreased toothbrushing frequency and RPI significantly mitigated this relationship. This highlights the impact of bullying on oral health and the protective influence of individual coping skills. Thus, the null hypothesis can be partially rejected.

Bullying perpetration and perpetration-victimization slightly increased untreated caries. This could be explained by the anti-social behavior and conduct problems associated with the perpetration psychology. Research showed that individuals with behavioral problems may adopt poor oral health practices, including decreased toothbrushing frequency [78], high sugar consumption [79] and poor attitude during dental visits [80]. This association did not reach statistical significance probably because life adversities have a dose-response relationship with oral health problems so their effect may not be detectable at this age, rather later in life [81]. The lack of association between bullying and gingival inflammation suggests a greater association with chronic oral conditions, as the longer an oral condition takes to develop, the more likely it will be affected by psychosocial factors [82]. There was a slight variation in gingivitis among the participants, which could make it challenging to explain this variation in the light of the studied variables [83]. Additionally, untreated caries, gingivitis and oral health-behavior are the product of multilevel influences. This would suggest that the psychological impact of bullying could have been counteracted or overshadowed by other individual, family or community-level influences [52].

The current findings are in line with previous studies reporting no significant association between bullying victimization and untreated caries in Nigerian adolescents [48] and dental problems in indigenous Australian adolescents [49]. On the other hand, the present findings disagree with previous studies showing significant associations between bullying victimization and untreated caries in Norwegian and Brazilian adolescents [47, 84] and gingival bleeding in Brazilian adolescents [85]. This disagreement could be attributed to the difference in the prevalence of bullying victimization, which was either too low (16% and 13%) [47, 85] or too high (78%) [84] as compared to (25%) in the present study. In addition, the personal perception of bullying influences the extent of its impact on mental health [86]. Indeed, some teachers in Egyptian schools would exercise verbal mortification and corporal punishment on students [87]. Hence, students may perceive verbal and physical violence from peers as the norm status, leaving a relatively milder impact on their mental status, consequently their oral health.

On the other hand, bullying victimization significantly reduced toothbrushing frequency. Victimized adolescents could brush their teeth less frequently because they may suffer from chronic stress, depression and low self-esteem [24]. Psychological distress may dominate an individual’s attention that they become less motivated to care for their oral hygiene [39]. Lower self-esteem was associated with less frequent toothbrushing [88]. Social problems could make adolescents less attentive to the social benefits of toothbrushing (clean teeth look attractive), driving them to neglect brushing their teeth [89]. This finding agrees with previous studies reporting less daily toothbrushing in victimized Norwegian adolescents [47] and Kuwaiti adolescents who did not feel accepted by their peers [90].

SOC and RPI per se were not associated with untreated caries, gingivitis or toothbrushing frequency. This could be because SOC forms during adolescence yet stabilizes around the age of thirty [53]. Unstable SOC may not have enabled the adolescents to fully overcome bullying, thereby control their oral health outcomes [74]. This disagrees with the literature supporting the significant association of SOC with favorable oral health and related behaviors [57, 91, 92]. Bell et al. [93] proposed that since adolescents have multiple friendship groups, resistance to one group of friends could be the result of conformity to another group. This would suggest that adolescents could still be influenced by their friends in making oral health decisions, despite having marked RPI. This disagrees with a previous study showing that weak RPI was significantly associated with higher risky health decisions among Chinese adolescents [94].

Regardless, the interaction of bullying perpetration, perpetration-victimization with SOC and RPI neutralized their impact on untreated caries, as well as the impact of bullying victimization and perpetration on toothbrushing frequency. This could be because higher SOC reduces school-related stress and depression [95, 96], and increases the feeling of well-being [53], which results in the adoption of more favorable toothbrushing habits [57]. This agrees with a previous study showing that SOC moderates the influence of racial discrimination on oral health-related quality of life in Brazilian adolescents [97]. Higher RPI is associated with higher resilience against life adversities [54, 98]. Students with greater resilience report experiencing less distress towards bullying victimization [99]. In addition, RPI regulates an individual’s social network [58], since one tends to select their friends based on similarities in behavior or attitudes [60]. Accordingly, victims befriend victims while bullies befriend bullies, and each are behaviorally influenced by each other [100], thus compounding the influence of bullying on oral health behaviors. However, a person with higher RPI could develop positive oral health practices, regardless of their friends’ behavior. This agrees with a previous study reporting a positive relationship between RPI and positive engagement in sports in adolescent athletes [54].

The current study showed several points of strength. First, it was conducted in schools, thereby capturing bullying at its most frequent setting [101]. Second, this study fully focused on adolescents unlike most studies which pooled adolescents with children of all ages [48, 102, 103], making it strenuous to discern these associations in adolescents. Third, the adolescents self-reported their own behaviors and psychosocial attributes which is more accurate than the parental or teacher proxy reporting. Fourth, the present study investigated the interaction of two coping skills (SOC and RPI) with bullying, revealing their protective effect. The present study, thus, fills a knowledge gap by providing evidence on the association between bullying, SOC and RPI and oral health in adolescents residing in a developing country, adding to the literature of psychosocial influences on oral health which mostly comes from developed countries [104, 105].

Despite its strengths, this study has some limitations. First, it was only conducted in public middle schools which could not capture bullying across various social levels or the trend of bullying throughout the entire period of adolescence. However, the present study aimed to capture bullying at its peak as bullying is more prevalent in public schools and culminates during middle school [106–109]. Second, the study examined bullying in schools only which could not include the out-of-school adolescents who represent more than one third of the adolescent population in North Africa and The Middle East [110]. Third, there was a high rate of absenteeism in schools because students depend on private tutoring for academic attainment [111], therefore a risk of selection bias exists as only students who attended school participated. This is compounded by victims’ tendency to not attend school [112], which indicates a possibly greater prevalence of bullying victimization. Fourth, self-reporting may be liable to both recall and social desirability biases. Both victim and bullies tend to underreport bullying, since victims refuse to show weakness [113] and bullies underestimate their aggressive behavior [114]. These measurement biases could lead to inaccurate estimates of associations, or over- or underestimation of risk parameters [115]. This was mitigated; however, by using an anonymous self-administered questionnaire, which made the adolescents more secure of the confidentiality of their responses. Fifth, the cross-sectional design, by its nature, cannot prove causality; therefore, longitudinal studies are needed to confirm such associations. Sixth, the lack of studies discussing the cross-cultural adaptation of the used scales could compromise cross-country comparisons of the study findings. However, this was mitigated through translating and back-translating the scales, modifying them to match the Egyptian culture, the assessment of the translated scales by a panel of experts and pilot-testing them on a sample of students [72].

The current study findings apply to adolescents of similar age in public schools. Caution is advised when generalizing to populations of different age in private schools or where the sociocultural context differs. This study results support the literature reporting the low to moderate prevalence and severity of dental caries, along with low frequency of toothbrushing in the Egyptian adolescent population [116, 117]. Hence, this needs to be taken into consideration when generalizing the current findings to populations with different oral health profiles where the impact of psychosocial factors would differ.

The study findings revealed the adverse impact of bullying victimization, perpetration and victimization-perpetration on oral health and showed the protective role of individual coping skills. Based on the current results, adolescents’ oral health could be enhanced through strengthening the laws and policies against bullying, integrating antibullying programs at schools, providing students with adequate mental health support, recognizing adolescents at risk and building their coping skills through effective training programs. Additionally, most anti-bullying programs tend to raise awareness on the dangers of bullying victimization only [118]; however, educating students on the health hazards associated with both bullying victimization and perpetration is urgently needed. Future longitudinal studies should assess bullying in a wider context which considers school climate, peers’ support and family dynamics.

## Conclusion

Although not statistically significant, bullying perpetration and perpetration-victimization were associated with higher untreated caries in Egyptian adolescents. Bullying victimization was significantly associated with lower frequency of toothbrushing and RPI significantly neutralized this relationship. Hence, future efforts aiming at enhancing the oral health of adolescents should focus on strengthening individual coping skills against life adversities.

## Electronic supplementary material

Below is the link to the electronic supplementary material.

Supplementary Material 1

## Data Availability

The dataset used and/or analyzed during the present study is available from the corresponding author on reasonable request.

## Abbreviations

SOC: Sense of Coherence
RPI: Resistance to Peer Influence
WHO: World Health Organization
UNESCO: United Nations Educational, Scientific and Cultural Organization
CAPMAS: Central Agency for Public Mobilization and Statistics
ICC: Intraclass correlation coefficient
GI: Gingival index
PLI: Plaque index
OBVQ-R: Revised Olweus Bully Victim Questionnaire
SOC-13: Sense of Coherence Scale Short Form
SD: Standard deviation
AOR: Adjusted odds ratio
CI: Confidence interval
B: Adjusted regression coefficient
SPSS: Statistical Package for Social Sciences
