# Supplementary material for: Bullying and oral health in Egyptian adolescents: the moderating role of sense of coherence and resistance to peer influence
Source: BMC Oral Health. 2024 Oct 18;24:1239. doi: 10.1186/s12903-024-04937-9 (PMC11490047; doi:10.1186/s12903-024-04937-9)
Supplement: Supplementary file 1 — Supplementary Material 1 [file 12903_2024_4937_MOESM1_ESM.docx]

**Appendix I**

The gingival condition was assessed using the gingival index of Löe and Silness (GI) [1] on 6 index teeth (#16, #12, #24, #36, #32, #44) and averaging the scores, which ranged from zero = no inflammation to 3 = severe inflammation, to obtain the adolescent’s GI score. Oral hygiene was measured by the plaque index of Silness and Löe (PLI) [2] on the same index teeth and averaging the scores, which ranged from zero = no plaque accumulation to 3 = abundant plaque, to obtain the adolescent’s score.

**References**

1. Löe H, Silness J. Periodontal Disease in Pregnancy. I. Prevalence and Severity. Acta Odontol Scand. 1963;21:533-51.

2. Silness J, Löe H. Periodontal Disease in Pregnancy. II. Correlation between Oral Hygiene and Periodontal Condtion. Acta Odontol Scand. 1964;22:121-35.
